# Irrelevance of Panton-Valentine leukocidin in hidradenitis suppurativa: results from a pilot, observational study

**DOI:** 10.1007/s10096-020-04002-7

**Published:** 2020-08-07

**Authors:** Monica Corazza, Alessandro Borghi, Vincenzo Bettoli, Roberto Pora, Ilaria Bononi, Elisa Mazzoni, Elisa Mazzola, Silva Saraceni, Martina Maritati, Carlo Contini

**Affiliations:** 1 Section of Dermatology and Infectious Diseases, Department of Medical Sciences, University of Ferrara, Via L. Ariosto 35, 44121 Ferrara, Italy; 2 Analysis Laboratory, S. Anna University Hospital, Ferrara, Italy; 3 Laboratories of Cell Biology and Molecular Genetics, Section of Pathology, Oncology and Experimental Biology, Department of Morphology, Surgery and Experimental Medicine, University of Ferrara, Ferrara, Italy; 4 Palermo, Italy

**Keywords:** Hidradenitis suppurativa, Panton-Valentine leukocidin, *Staphylococcus aureus*, Infections, RT-qPCR

## Abstract

Panton-Valentine leukocidin (PVL) appears to be a virulence factor which, among others, can exacerbate the pathogenicity of *Staphylococcus aureus* infections, especially inducing severe necrotic, deep-seated skin infections, abscesses, and recurrences. These peculiarities have some overlaps with hidradenitis suppurativa (HS). Our main aim was to assess if *S. aureus* producing PVL could have some role in influencing clinical features and/or course of HS, specifically in the suppuration and recurrence of lesions. This pilot, mono-centric, observational study included all adult subjects affected with HS consecutively referring to our HS clinic over a 3-month period. Clinically evident suppuration and at least 2 weeks wash out from any antibiotic were the main inclusion criteria. Purulent material from HS skin lesions was collected with swabs in order to isolate micro-organisms, with specific regard to *S. aureus.* Detection of PVL was performed by real-time quantitative PCR (RT-qPCR). We also analyzed purulent material from suppurative skin lesions other than HS, as a control. Thirty HS patients were included; 29 purulent lesions (96.7%) harbored at least one bacterial species. Five (16.7%) swab samples were positive for *S. aureus*, none of which was positive for PVL genes. Among the 30 purulent disorders included as controls, 8 (26.3%) were positive for *S. aureus*; of these, 4 strains (50%) expressed LPV. The study results seem to exclude the pathogenetic involvement of *S. aureus* producing PVL in HS; as a result, PVL does not seem to represent a potential target in the future development of HS treatments.

## Introduction

Hidradenitis suppurativa (HS), also known as acne inversa, is a chronic-relapsing, debilitating inflammatory disease of the hair follicle, which affects apocrine gland-bearing skin, most commonly the axillae, inguinal regions, anogenital area, and infra- and inter-mammary folds [1]. It is clinically characterized by recurrent, painful, deep-seated nodules commonly ending in abscesses and sinus tracts with persistent suppuration and hypertrophic, bridged scarring. HS usually presents after puberty and its estimated prevalence ranges from less than 0.1 to 4% [1, 2]. HS has a huge negative influence on patients’ lives due to its chronic course, pain and other disease-related symptoms, persistent malodor, peculiar anatomical localization, disfiguring sequelae, and lack of a definitive cure [3, 4].

The pathogenesis of HS is not fully recognized and appears to involve a number of interacting factors, including a susceptible genetic background, a dysregulated immune response to various heterogeneous stimuli, and follicular occlusion [1, 2, 5]. The role of bacterial infection in the initiation or propagation of HS remains under investigation [6, 7]. Most frequently bacteriological studies have shown a mixed growth of commensal microbes, which is more consistent with the view that HS is an immune-mediated condition rather than a disease of primary infectious etiology [7, 8]. Bacterial colonization of HS lesions may act synergistically with a dysfunctional immune response, involving both the innate and adaptive immune systems, which leads to a chronic, relapsing inflammatory scenario. On the other hand, HS may predispose to an increased risk of skin infections [9]. Panton-Valentine leukocidin (PVL) is a two-component pore-forming cytotoxin produced by *Staphylococcus aureus* inducing leukocyte destruction and tissue necrosis [10]. In addition, PVL is a polymorphonuclear neutrophils priming agent [11]. In keeping with this, it appears to be a highly relevant virulence factor which, among others, can exacerbate the pathogenicity of *S. aureus* infections [12]. In particular, it is related to deep-seated skin and soft tissue infections and causes furuncles, cutaneous abscesses, and severe necrotic skin infections [13, 14]. Moreover, PVL seems to be a marker for severity and recurrence [15]. Based on these assumptions, we were interested in assessing if *S. aureus* producing PVL could have some role in influencing clinical features and/or the course of HS, especially in the suppuration and recurrence of lesions.

## Materials and methods

### Study design and patients

In the present pilot, mono-centric, observational study, we included all adult subjects affected by HS consecutively referring to our HS clinic over a 3-month period. Patients were excluded in the presence of the following: (i) lack of reliable diagnosis, (ii) absence of clinically evident suppuration, (iii) topical or systemic antibiotics during the 2 weeks before inclusion. Purulent material from HS skin lesions was collected with swabs in order to isolate micro-organisms, with specific regard to *S. aureus* releasing PVL. We also collected purulent material from suppurative skin lesions other than HS, over a 3-month period, as a control. Controls were excluded if they were treated with topical and/or systemic antibiotics or if they had a diagnosis of HS or clinical features resembling HS. The eligible subjects who were visited more than once over the study period underwent skin swabs only at the first visit.

This was a spontaneous survey, with no funding from external sources. The study was approved by the University-Hospital of Ferrara institutional review board. Both patients and controls provided their written informed consent.

### Data collection

The following data from HS patients were recorded by using a standardized data collection form: (1) age at inclusion; (2) gender; (3) disease duration, taken as the time between the patient-reported onset of symptoms and/or signs and study inclusion; (4) previous treatments, defined as documented courses with any pharmacological active or surgical interventions prior to entering this study; (5) current treatment; (6) Hurley stage; (7) sites involved by the disease; (8) number of sites with active suppuration at study inclusion; (9) number of suppurative episodes during the previous 6 months; (10) isolation of microbes, in particular *S. aureus*; (11) Panton-Valentine leukocidin expression by *S. aureus* strains.

From controls, we recorded the following information: (1) diagnosis of purulent skin disorders they were affected by; (2) isolation of *S. aureus*; (3) Panton-Valentine leukocidin expression. These purulent skin disorders were categorized as follows: (i) primary pyodermitis versus secondary pyodermitis/impetiginization of another primary skin disorder; (ii) primarily follicular versus non-follicular purulent disorders; (iii) deep versus superficial inflammatory process.

### Microbiological assessments

Samples were collected from the purulent material from HS draining lesions as well as from control suppurative disorders. One sample was collected from a single purulent lesion of each included subject. Content was drained with gentle pressure exercised on previously sterilized skin surface, using sterile gloves and swabs. A suitable transport media for aerobes and anaerobes was used (ESwab Liquid Amies Collection and Preservation System, Catalog No. 490CE.A, Copan Diagnostic, Murrieta, CA, USA). Specimens were stored and delivered to the laboratory according to the manufacturer’s instructions.

Swabs from clinical specimens were grown on several selective agar plates to isolate specific organisms, as described above [16]. The *S. aureus* culture suspensions were then stored at 4 °C prior to nucleic acid extraction and PCR analysis. The commercial kit RIDA®GENE PVL assay was used for the extraction of bacterial DNA and for the real-time quantitative PCR (RT-qPCR) analysis through the amplification of a PVL-specific fragment (Panton Valentine Leukocidine lukF-PV). To verify whether cross-contamination had occurred during DNA extraction, purification, and PCR procedures, each sample was processed simultaneously with negative controls, represented by sample lacking DNA (real-time PCR mix). Samples and controls were analyzed in duplicate replica experiments. Samples were run in CFX96 Touch Real-Time PCR Detection System (Applied Biosystems) instrument and sample analysis was performed by Bio-Rad CFX Manager software, following the manufacturer’s instructions. Positive and negative controls must show correct results. The positive control had a concentration of 10^3^ copies/μl. A total of 5 × 10^3^ copies were used in each RT-qPCR run. The RIDA®GENE PVL detection limit was ≤ 5 copies of DNA for the reaction.

### Statistics

Continuous variables were presented as mean ± standard deviation (SD) and categorical variables as frequencies and percentages. Comparisons between groups were made using *t* test for quantitative variables, while Pearson’s chi-squared test or the Fisher exact test was used for qualitative variables. For categorical data, odds ratios (OR) with 95% confidence intervals (95% CI) were calculated. All statistical analyses were performed using Stata version 13, setting the significant level *α* to 0.05.

## Results

### HS patients

Thirty patients affected by HS were included. Table 1 reports the main HS patients’ characteristics. The majority of patients (19 of 30, 63.3%) had a Hurley stage III, 16 (53.3%) had at least 3 different anatomical sites involved by HS, and 9 (30%) reported ≥ 6 episodes of suppuration during the previous 6 months. Seventeen patients (56.7%) had undergone at least 3 previous different treatments and 20 (66.7%) were in treatment with drugs other than antibiotics.

**Table 1:** Main characteristics of the patients affected with hidradenitis suppurativa

	Total	Males	Females
Patients, *n* (%)	30 (100%)	21 (70%)	9 (30%)
Age, mean ± SD	33.5 ± 15.05	32 ± 12.7	38 ± 18.23
Disease duration, years, mean ± SD	11.5 ± 10.01	10 ± 9.8	15 ± 9.79
Previous treatments	30 (100%)	21 (100%)	9 (100%)
Systemic antibiotics, *n* (%)	30 (100%)	21 (100%)	9 (100%)
Topical antibiotics, *n* (%)	16 (53%)	11 (52%)	5 (56%)
Surgery, *n* (%)	9 (30%)	7 (33%)	2 (22%)
Biologics, *n* (%)	7 (23%)	6 (29%)	1 (11%)
Zinc derivatives, *n* (%)	7 (23%)	5 (24%)	2 (22%)
Systemic retinoids, *n* (%)	6 (20%)	5 (24%)	1 (11%)
Dapsone, *n* (%)	6 (20%)	4 (19%)	2 (22%)
Systemic corticosteroids, *n* (%)	2 (7%)	1 (5%)	1 (11%)
Cyclosporine, *n* (%)	1 (3%)	1 (5%)	0
One previous treatment, *n* (%)	5 (16%)	4 (19%)	1 (11%)
Two previous treatments, *n* (%)	8 (27%)	4 (19%)	4 (44%)
Three or more previous treatments, *n* (%)	17 (57%)	13 (62%)	4 (44%)
Current treatments	20 (67%)	15 (71%)	5 (56%)
Biologics, *n* (%)	15 (50%)	11 (52%)	4 (44%)
Zinc derivatives, *n* (%)	3 (10%)	3 (14%)	0
Systemic retinoids, *n* (%)	1 (3%)	1 (5%)	0
Cyclosporine, *n* (%)	1 (3%)	0	1 (11%)
Hurley stage			
I, *n* (%)	3 (10%)	2 (10%)	1 (11%)
II, *n* (%)	8 (27%)	7 (33%)	1 (11%)
III, *n* (%)	19 (63%)	12 (57%)	7 (78%)
Sites involved*, mean SD	2.8 ± 1.3	2.8 ± 1.4	2.8 ± 1
Axillae	25 (83%)	20 (95%)	5 (56%)
Inguinal and anogenital areas	23 (77%)	14 (67%)	9 (100%)
Buttocks	9 (30%)	7 (33%)	2 (22%)
Abdomen	5 (17%)	4 (19%)	1 (11%)
Infra- and inter-mammary folds	3 (10%)	1 (5%)	2 (22%)
Back	2 (7%)	2 (10%)	0
Inner thighs	1 (3%)	1 (5%)	0
One site, *n* (%)	6 (20%)	5 (24%)	1 (11%)
Two sites, *n* (%)	8 (27%)	5 (24%)	3 (33%)
Three or more sites, *n* (%)	16 (53%)	11 (52%)	5 (56%)
Number of sites with active suppuration, mean ± SD	1.5 ± 0.7	1.4 ± 0.73	2.4 ± 1
Number of suppurative episodes during the previous 6 months, mean ± SD	5 ± 5.6	5.7 ± 6.3	5.4 ± 4.3
Isolation of *S. aureus*, *n* (%)	5 (17%)	3 (14%)	2 (22%)
Panton-Valentine leukocidin positivity, *n* (%)	0	0	0

*Patient could have simultaneously more than one anatomical site involved by HS

All HS samples but one harbored at least one bacterial species (detailed in Table 2). Five (16.7%) swab samples were positive for *S. aureus*. Isolation of *S. aureus* did not significantly correlate with disease Hurley stage (Hurley stage III had an OR 2.6667, 95% CI: 0.2587 to 27.4865, *p* = 0.409), gender (males had an OR 0.5833, 95% CI: 0.0797 to 4.2711, *p* = 0.5957), previous systemic antibiotics (OR 0.2157, 95% CI: 0.0038 to 12.0965, *p* = 0.4553), patient age (*p* = 0.11), number of involved sites (*p* = 0.78), number of current suppurative lesions (*p* = 0.44), and number of suppurative recurrences during the previous 6 months (*p* = 0.15). Disease duration was higher (18.6 years, SD ± 11.80) among patients harboring *S. aureus* than among the others (10.1, SD ± 9.28) (*p* = 0.04). None of the *S. aureus* strains isolated was positive for PVL genes.

**Table 2:** Bacterial isolates from purulent material drained from hidradenitis suppurativa lesions, other than *S. aureus*

Bacteria	Number (%)
Coagulase-negative staphylococci	16 (53%)
*S. epidermidis*	7 (23%)
*S. haemoliticus*	7 (23%)
*S. lugdunensis*	2 (7%)
Peptostreptococcaceae	8 (27%)
*P. anaerobius*	5 (17%)
*P. asaccharolyticus*	1 (3%)
*F. magna*	2 (7%)
Enterobacteriaceae	9 (30%)
*P. mirabilis*	6 (20%)
*E. coli*	2 (7%)
*C. diversus*	1 (3%)
*S. anginosus*	2 (7%)
*P. bivia*	2 (7%)
*E. faecalis*	1 (3%)
*C. striatum*	1 (3%)
*P. aeruginosa*	1 (3%)
*A. turicensis*	1 (3%)

### Controls

Table 3 shows clinical diagnosis and swab sample data of the 30 purulent disorders included as controls (17 males and 13 females, mean age 39 ± 18.59). Among these samples, 8 (26.3%) were positive for *S. aureus*. Four *S. aureus* strains (50%) expressed PVL. Expression of PVL was strictly related with primary pyodermitis (OR 3.5000, 95% CI: 0.3204 to 38.2337, *p* = 0.3044), primarily follicular inflammations (OR 10.4400, 95% CI: 0.5104 to 213.5310, *p* = 0.127), and deep infectious processes (OR 4.2000, 95% CI: 0.4704 to 37.5001, *p* = 0.198). None of these associations reached the significance threshold, probably due to the small sample sizes.

**Table 3:** Purulent skin disorders included as controls

Skin disorder	Number (%)	Isolation of *S. aureus*, *n* (% of each disorder)	Panton-Valentine leukocidin expression, *n* (% of each disorder)
Folliculitis, both superficial and deep	14 (47%)	5 (36%)	3 (21%)
Erysipelas	1 (3%)	0	0
Pustules in acne vulgaris or rosacea	5 (17%)	0	0
Paronychia	2 (6%)	1 (50%)	0
Folliculitis decalvans	2 (6%)	2 (100%)	1 (50%)
Purulent secondary pyodermitis/impetiginizations	6 (20%)	0	0
Subgroups			
Primary pyodermitis	15 (50%)	5 (30%)	3 (20%)
Follicular purulent disorders	16 (53%)	7 (44%)	4 (25%)
Deep inflammation	7 (23%)	3 (43%)	2 (29%)

## Discussion

Bacteria have long been considered in the pathogenesis of HS. It is generally acknowledged that bacteria do not play a direct role in the etiology of HS; however, they may be involved in the pathogenesis of HS via follicular dysbiosis and biofilm as well as eliciting an inflammatory response in a genetically predisposing background [7]. Interestingly, it had previously been shown that the microbiological population of HS changes considerably according to the clinical severity of the disease [17]. Our findings support the high prevalence of positive culturing samples from HS lesions [7], since bacteria specimens were isolated from almost all the study samples (29 of 30, 96.7%).

With special reference to *S. aureus*, this has often been described in association with HS and has even been proposed as a potential causative organism [18, 19]. However, the literature includes some controversial data [20] and its action in the pathogenesis of the disease remains unknown. Based on the recognized virulent role of PVL in *S. aureus* infections, especially in those by community-associated methicillin-resistant *S. aureus* (CA-MRSA), also at skin level [21, 22], we wanted to specifically seek its presence in HS lesions. In particular, we were interested in deepening a possible link between *S. aureus* producing PVL and degree of suppuration, skin involvement, disease chronicity, and recurrence. We focused on PVL because it exerts peculiar mechanisms of action that may be in some way compatible with HS. In fact, it induces the release of pro-inflammatory cytokines and nuclear factor kappa B (NF-κB) in neutrophils, leading to skin infections which tend to be invasive, necrotizing, and recurrent [22, 23]. Analyzing the findings obtained from the purulent skin disorders included as controls, these were fairly consistent with this pathogenic profile (Table 3). Prevalence of PVL-positive *S. aureus* was highly correlated with primary pyodermitis when compared with impetiginized lesions, primarily follicular inflammation and deep inflammatory processes.

The rate of *S. aureus* strains isolated from purulent material drained from abscesses and sinuses of HS patients was quite in line with previous reports, being about 17% [7, 19, 24]. The isolation of *S. aureus* did not correlate with patient or disease characteristics, including severity of HS in terms of Hurley’s score, number of sites affected, and frequency of recurrence, unlike what other researchers have found [25]. The most noteworthy finding of our study was that none of the strains of *S. aureus* isolated from HS purulent lesions expressed PVL. This finding strongly suggests an irrelevant role of PVL, not only in HS etiopathogenesis but also in its clinical features and course. Therefore, in speculating about the relevance of *S. aureus* in HS, our results seem to exclude the involvement of PVL. This can also be seen from a therapeutic perspective, since PVL does not seem to represent a potential target in the development of a treatment for HS.

The role of toxins, especially necrotizing toxins, in HS is uncertain and has been poorly investigated. Diphtheria by the toxigenic zoonotic pathogen *Corynebacterium ulcerans* is increasingly occurring in children, adolescents, and adults. In addition to diphtheria toxin (DT), the exotoxin phospholipase D (PLD) is considered an important virulence factor of *C. ulcerans* and *C. pseudotuberculosis* [26]. PLD has been found to induce dermonecrotic lesions, increased vascular permeability in vivo, and synergistic hemolysis of sheep blood cells.

*Pseudomonas aeruginosa* can cause severe human opportunistic infections, including many hospital-acquired infections. However, there is scarce evidence about its clinical relevance in patients with HS. Among its toxins, exotoxin A is the main virulence factor [27]. Similarly but less effectively than DT, exotoxin A interrupts protein synthesis in the host cell and has an immunosuppressive action. Pigments, such as pyocyanin, which catalyzes reactive oxygen species (ROS) production and attracts neutrophils, and pioverdin (siderophore), exert multiple detrimental effects on the host too. The blue-green phenazine, pyocyanin, is a key virulence factor that can kill competing organisms as well as host cells and can inactivate catalases to protect against ROS generated by host tissues [28]. Pyocyanin in particular can serve as a redox cycler similar to methyl viologen, resulting in superoxide radical production and oxidative stress [29]. The majority of these virulence factors are under the control of two regulatory systems: the two-component system and the sensing quorum, which allow the survival and multiplication of this microorganism in the host [30].

Despite the inherent potential to cause harmful effects in humans, little is known about the role of these toxins in HS and further investigation could be conducted. Furthermore, the methods used for their detection will still require time for their optimal development and application in this specific context.

This study has some limitations, especially the relatively small number of patients affected with HS included. However, it was designed as pilot in nature and with the possibility of an extension being planned in the event of positive results. The patients attended a tertiary clinic, specifically dedicated to HS, so the study population may be not representative of the entire population affected by this disease and a selection bias cannot be excluded. Other variables, such as comorbidities, body mass index, and lifestyle habits like smoking, potentially relevant for the study assessments, were not considered.

In spite of these limitations, this is the first study to address the potential role of an *S. aureus* virulence factor, like PVL, in HS clinical expression. The results of our study seem to exclude its pathogenetic involvement as well as its relevance in the management of patients.

## Data Availability

All data generated or analyzed during this study are included in this manuscript. Detailed data are available from the corresponding author on request.

## References

[1] Zouboulis CC, Bechara FG, Dickinson-Blok JL, Gulliver W, Horváth B, Hughes R, Kimball AB, Kirby B, Martorell A, Podda M, Prens EP, Ring HC, Tzellos T, van der Zee HH, van Straalen KR, Vossen ARJV, Jemec GBE (2019). Hidradenitis suppurativa/acne inversa: a practical framework for treatment optimization - systematic review and recommendations from the HS ALLIANCE working group. J Eur Acad Dermatol Venereol.

[2] Goldburg SR, Strober BE, Payette MJ (2020). Part I. Hidradenitis suppurativa: epidemiology, clinical presentation, and pathogenesis. J Am Acad Dermatol.

[3] Jørgensen AR, Holm JG, Ghazanfar MN, Yao Y, Ring HC, Thomsen SF (2019) Factors affecting quality of life in patients with hidradenitis suppurativa. Arch Dermatol Res. 10.1007/s00403-019-02025-5 10.1007/s00403-019-02025-5 31848682

[4] Matusiak Ł (2018) Profound consequences of hidradenitis suppurativa: a review. Br J Dermatol. 10.1111/bjd.16603 10.1111/bjd.16603 29744872

[5] Tricarico PM, Boniotto M, Genovese G, Zouboulis CC, Marzano AV, Crovella S (2019). An integrated approach to unravel hidradenitis suppurativa etiopathogenesis. Front Immunol.

[6] Satoh TK, Mellett M, Contassot E, French LE (2018). Are neutrophilic dermatoses autoinflammatory disorders?. Br J Dermatol.

[7] Ring HC, Riis Mikkelsen P, Miller IM, Jenssen H, Fuursted K, Saunte DM, Jemec GB (2015). The bacteriology of hidradenitis suppurativa: a systematic review. Exp Dermatol.

[8] Nikolakis G, Join-Lambert O, Karagiannidis I, Guet-Revillet H, Zouboulis CC, Nassif A (2015). Bacteriology of hidradenitis suppurativa/acne inversa: a review. J Am Acad Dermatol.

[9] Lee HH, Patel KR, Singam V, Rastogi S, Silverberg JI (2020). Associations of cutaneous and extracutaneous infections with hidradenitis suppurativa in U.S. children and adults. Br J Dermatol.

[10] Genestier AL, Michalete MC, Prévoset G, Bellot G, Chalabreysse L, Peyrol S, Thivolet F, Etienne J, Lina G, Vallette FM, Vandenesch F, Genestier L (2005). Staphylococcus aureus Panton-Valentine leucocidin directly targets mitochondria and induces Bax-independent apoptosis of buman neutrophils. J Clin Invest.

[11] Colin DA, Monteil H (2003). Control of the oxidative burst of human neutrophils by staphylococcal leukotoxins. Infect Immun.

[12] Vandenesch F, Naimi T, Enright MC, Lina G, Nimmo GR, Heffernan H, Liassine N, Bes M, Greenland T, Reverdy ME, Etienne J (2003). Community acquired methicillin-resistant Staphylococcus aureus carrying Panton-Valentine leukocidin genes: worldwide emergence. Emerg Infect Dis.

[13] Varshney AK, Martinez LR, Hamilton SM, Bryant AE, Levi MH, Gialanella P, Stevens DL, Fries BC (2010). Augmented production of Panton-Valentine leukocidin toxin in methicillin-resistant and methicillin-susceptible Staphylococcus aureus is associated with worse outcome in a murine skin infection model. J Infect Dis.

[14] Klein S, Menz MD, Zanger P, Heeg K, Nurjadi D (2019). Increase in the prevalence of Panton-Valentine leukocidin and clonal shift in community-onset methicillin-resistant Staphylococcus aureus causing skin and soft-tissue infections in the Rhine-Neckar Region, Germany, 2012-2016. Int J Antimicrob Agents.

[15] Zetola N, Francis JS, Nuermberger EL, González MPM, Limon E, Hornero A, Martín R, Gudiol F, Ariza J (2005). Community-acquired methicillin-resistant Staphylococcus aureus: an emerging threat. Lancet Infect Dis.

[16] Bettoli V, Manfredini M, Massoli L, Carillo C, Barozzi A, Amendolagine G, Ruina G, Musmeci D, Libanore M, Curtolo A, Mantovani L, Contini C, Pellacani G, Corazza M (2019). Rates of antibiotic resistance/sensitivity in bacterial cultures of hidradenitis suppurativa patients. J Eur Acad Dermatol Venereol.

[17] Guet-Revillet H, Jais J-P, Ungeheuer M-N, Coignard-Biehler H, Duchatelet S, Delage M, Lam T, Hovnanian A, Lortholary O, Nassif X, Nassif A, Join-Lambert O (2017). The microbiological landscape of anaerobic infections in hidradenitis suppurativa: a prospective metagenomic study. Clin Infect Dis.

[18] Lapins J, Jarstrand C, Emtestam L (1999). Coagulase-negative staphylococci are the most common bacteria found in cultures from the deep portions of hidradenitis suppurativa lesions, as obtained by carbon dioxide laser surgery. Br J Dermatol.

[19] Jemec GB, Faber M, Gutschik E, Wendelboe P (1996). The bacteriology of hidradenitis suppurativa. Dermatology.

[20] Sartorius K, Killasli H, Oprica C, Sullivan A, Lapins J (2012). Bacteriology of hidradenitis suppurativa exacerbations and deep tissue cultures obtained during carbon dioxide laser treatment. Br J Dermatol.

[21] Hanawa T, Shimoda-Komatsu Y, Araki K, Ohyama M, Ohnishi H, Kamiya S, Matsuda T (2020). Skin and soft tissue infections caused by different genotypes of PVL-positive community-acquired methicillin-resistant Staphylococcus aureus strains. Jpn J Infect Dis.

[22] Saeed K, Gould I, Esposito S, Ahmad-Saeed N, Ahmed SS, Alp E, Bal AM, Bassetti M, Bonnet E, Chan M, Coombs G, Dancer SJ, David MZ, De Simone G, Dryden M, Guardabassi L, Hanitsch LG, Hijazi K, Krüger R, Lee A, Leistner R, Pagliano P, Righi E, Schneider-Burrus S, Skov RL, Tattevin P, Van Wamel W, Vos MC, Voss A, International Society of Chemotherapy (2018). Panton-Valentine leukocidin-positive Staphylococcus aureus: a position statement from the International Society of Chemotherapy. Int J Antimicrob Agents.

[23] Shallcross LJ, Fragaszy E, Johnson AM, Hayward AC (2013). The role of the Panton-Valentine leucocidin toxin in staphylococcal disease: a systematic review and meta-analysis. Lancet Infect Dis.

[24] Guet-Revillet H, Coignard-Biehler H, Jais JP, Quesne G, Frapy R, Poirée S, Le Guern AS, Le Flèche-Matéos A, Hovnanian A, Consigny PH, Lortholary O, Nassif X, Nassif A, Join-Lambert O (2014). Bacterial pathogens associated with hidradenitis suppurativa, France. Emerg Infect Dis.

[25] Katoulis A, Koumaki V, Efthymiou O, Koumaki D, Dimitroulia E, Voudouri M, Voudouri A, Bozi E, Tsakris A (2020). Staphylococcus aureus carriage status in patients with hidradenitis suppurativa: an observational cohort study in a tertiary referral hospital in Athens, Greece. Dermatology.

[26] Simpson-Lourêdo L, Silva CMF, Hacker E, Souza NF, Santana MM, Antunes CA, Nagao PE, Hirata R, Burkovski A, Villas Bôas MHS, Mattos-Guaraldi AL (2019). Detection and virulence potential of a phospholipase D-negative Corynebacterium ulcerans from a concurrent diphtheria and infectious mononucleosis case. Antonie Van Leeuwenhoek.

[27] Crousilles A, Maunders E, Bartlett S, Fan C, Ukor EF, Abdelhamid Y, Baker Y, Floto A, Spring DR, Welch M (2015). Which microbial factors really are important in Pseudomonas aeruginosa infections?. Future Microbiol.

[28] Wilson R, Sykes DA, Watson D, Rutman A, Taylor GW, Cole PJ (1988). Measurement of Pseudomonas aeruginosa phenazine pigments in sputum and assessment of their contribution to sputum sol toxicity for respiratory epithelium. Infect Immun.

[29] Van Laar TA, Esani S, Birges TJ, Hazen B, Thomas JM, Rawat M (2018). Pseudomonas aeruginosa gshA mutant is defective in biofilm formation, swarming, and pyocyanin production. mSphere.

[30] Ben Haj Khalifa A, Moissenet D, Vu Thien H, Khedher M (2011). Virulence factors in Pseudomonas aeruginosa: mechanisms and modes of regulation. Ann Biol Clin (Paris).

